# Classification of first-episode schizophrenia patients, individuals at ultra-high risk for psychosis, and healthy controls using structural mri, eeg, and machine learning

**DOI:** 10.1192/j.eurpsy.2021.1097

**Published:** 2021-08-13

**Authors:** A. Tomyshev, N. Lutsyak, M. Belyaev, V. Kaleda, I. Lebedeva

**Affiliations:** 1 Laboratory Of neuroimaging And Multimodal Analysis, FSBSI Mental Health Research Center, Moscow, Russian Federation; 2 Laboratory Of Data Analysis And Predictive Modeling, Institute for Information Transmission Problems of the Russian Academy of Sciences (Kharkevich Institute), Moscow, Russian Federation; 3 Center For Neurobiology And Brain Restoration, Skolkovo Institute of Science and Technology, Moscow, Russian Federation; 4 Department Of Endogenous Mental Disorders, FSBSI Mental Health Research Center, Moscow, Russian Federation

**Keywords:** machine learning, psychosis, MRI, EEG

## Abstract

**Introduction:**

Machine learning has increasingly been applied to classification of psychosis spectrum in neuroimaging research. However, a number of multimodal studies using MRI and electroencephalography (EEG) is quite limited.

**Objectives:**

To assess the power of multimodal structural MRI (sMRI) and EEG data to provide pairwise discrimination between first-episode schizophrenia (FES) patients, individuals at ultra-high-risk of psychosis (UHR), and healthy controls (HC) using machine learning algorithms.

**Methods:**

46 FES male patients, 39 UHR individuals, and 54 matched HC underwent sMRI (3T Philips scanner) and electroencephalography. T1-weighted images were processed via FreeSurfer to obtain cortical and subcortical measures. L2 regularized logistic regression was used to evaluate the efficacy of diagnostic prediction.

**Results:**

The accuracies of pairwise discriminations were: 87% for FES vs HC (specificity 83%, sensitivity 91%); 77% for FES vs UHR (specificity 76%, sensitivity 79%); 75% for UHR vs HC (specificity 77%, sensitivity 73%).

**Conclusions:**

Current findings suggest that the patterns of anatomical and functional variability have potential as biomarkers for discrimination between schizophrenia, UHR, and healthy subjects. Furthermore, results show that the selection and multimodality of feature types are important. Specifically, adding EEG data to morphometric measures improved accuracy rates in FES vs HC and FES vs UHR contrasts, whereas standalone EEG data provided higher accuracy compared with morphometric or multimodal data in UHR vs HC discrimination. Expectedly, predictive power for the UHR was smaller than for the FES due to its intermediate anatomical features, located between those observed in healthy controls and those found in patients. The work was supported by RFBR grant 20-013-00748

